# Combining Computational Fluid Dynamics and Agent-Based Modeling: A New Approach to Evacuation Planning

**DOI:** 10.1371/journal.pone.0020139

**Published:** 2011-05-31

**Authors:** Joshua M. Epstein, Ramesh Pankajakshan, Ross A. Hammond

**Affiliations:** 1 Center for Advanced Modeling, Department of Emergency Medicine, The Johns Hopkins University, Baltimore, Maryland, United States of America; 2 External Faculty, Santa Fe Institute, Santa Fe, New Mexico, United States of America; 3 Graduate School of Public Health, University of Pittsburgh, Pittsburgh, Pennsylvania, United States of America; 4 SimCenter: National Center for Computational Engineering, University of Tennessee, Chattanooga, Tennessee, United States of America; 5 Center on Social Dynamics and Policy, Economic Studies Program, The Brookings Institution, Washington D.C., United States of America; Indiana University, United States of America

## Abstract

We introduce a novel hybrid of two fields—Computational Fluid Dynamics (CFD) and Agent-Based Modeling (ABM)—as a powerful new technique for urban evacuation planning. CFD is a predominant technique for modeling airborne transport of contaminants, while ABM is a powerful approach for modeling social dynamics in populations of adaptive individuals. The hybrid CFD-ABM method is capable of simulating how large, spatially-distributed populations might respond to a physically realistic contaminant plume. We demonstrate the overall feasibility of CFD-ABM evacuation design, using the case of a hypothetical aerosol release in Los Angeles to explore potential effectiveness of various policy regimes. We conclude by arguing that this new approach can be powerfully applied to arbitrary population centers, offering an unprecedented preparedness and catastrophic event response tool.

## Introduction

We introduce a novel hybrid of two fields—Computational Fluid Dynamics (CFD) and Agent-Based Modeling (ABM)—as a powerful technique for risk assessment, preparedness, and response for a spectrum of hazards. CFD is the most accurate technique for modeling airborne transport of contaminants [1]–[3]. ABM is an especially powerful technique for modeling populations of adaptive individuals interacting with one another and with their environment [4]–[5]. Each field has developed rapidly, but independently, as computing power has increased. The hybrid CFD-ABM method introduced here synthesizes the two, simulating how large spatially-distributed populations of agents might respond in the face of a physically realistic contaminant plume. In this “proof-of-concept” exposition, we demonstrate the feasibility of CFD-ABM evacuation design, using the case of an aerosol release in Los Angeles. Computational representation of LA buildings, occupancy, and infrastructure are based on commercially-available LIDAR data and USGS street-level data [3]. We use this hybrid approach to estimate aerosol exposure levels for various cases. In the base (no mitigation) case, mass evacuation of buildings quickly floods egress routes producing high traffic congestion and high levels of exposure. We explore two parameters: shelter-in-place compliance and building permeability (modeled as a scale factor for the local contaminant concentration). Pronounced nonlinear thresholds obtain, but substantial improvement over base-case outcome can be achieved through combinations of interventions. Behavioral considerations—levels of compliance—strongly affect the efficacy of policy. We argue that the general approach can be powerfully applied to arbitrary population centers, offering an unprecedented preparedness and catastrophic event response tool.

The CFD modeling was done using the *Tenasi* Computational Fluid Dynamics suite, which solves the Reynolds averaged Navier-Stokes (RANS) equations on general unstructured grids using a node-centered finite volume implicit scheme with 2^nd^ order temporal and 3^rd^ order spatial discretizations. The statically load balanced domain decomposition parallel solution algorithm is scalable on thousands of processors. The simulations in this study used a preconditioned RANS/DES solver, called the arbitrary Mach solver [1], which is capable of handling flows ranging from the low subsonic (Mach number <0.001) to the supersonic (Mach number >1). The formulation also allows the accurate simulation of buoyancy effects at low speeds. A scalar transport equation with diffusion terms was solved to simulate aerosol dispersal. A Detached Eddy Simulation (DES) [2] approach was used to avoid known weaknesses of RANS for heavily separated flows.

The agent-based model (ABM) is a new scientific instrument made possible by advances in computing [4]–[5]. An ABM includes a population of agents, typically representing individual humans, each implemented as a distinct data structure in a computer program. These agents interact locally in a relevant space (here, Los Angeles). Over many iterations, these micro-interactions generate large scale macroscopic regularities (e.g., spatial disease patterns, traffic patterns) that can in principle be directly compared to data, permitting empirical corroboration, as has been done in other agent-based models [6]–[7]. In ABMs, populations are *heterogeneous*; individuals may differ in myriad ways—by workplace, disease susceptibility, behavioral rules—all of which may evolve over time through interactions with other agents and the environment. Paramount for behavioral modeling is that real people—unlike *homo economicus*—do not have perfect information, and do not optimize. They exhibit *bounded rationality* [8], using simple heuristics, norms, biased and partial reporting, and peer behaviors in their decision-making. Both laboratory social science and empirical field research support the bounded rationality hypothesis [9]. Agent models accommodate this spatial and behavioral realism in ways that competing approaches cannot, and can do so *transparently*. Agents are implemented in C++ as instantiations of two base classes, one for agents within buildings and the second for agents on the road.

## Methods

As a concrete illustration of the hybrid CFD-ABM approach, we develop a model based on downtown Los Angeles. The area modeled is approximately 2.2 square miles, in a rectangle from (UTM Zone 11 0382395E, 3767104N) to (UTM Zone 11 0385887E, 3769069N).

*Physical Layout.* Building data consisting of 2000 structures was extracted from the CFD grid^3^ and merged with street maps from the US Geological Survey [10]. Lane information is adapted from Google maps for freeways, and is assumed to be four lanes for surface streets.

*Occupancy.* The simulation begins with people (agents) inside buildings and vehicles on the streets. The number of people inside *each* building is estimated using information from the CFD grid geometry and from the office planning literature [11]. The number of occupants (N) in building i is:



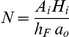
where the height (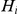) and floor area (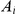) for each building are taken from the CFD grid and standard height-per-floor (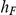) and area per occupant (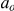) are constants taken from the literature.

*Traffic.* The simulation is started with 20000 vehicles on the streets. Pedestrians are not modeled. Once people exit buildings, they are assumed to enter cars which can accumulate in the surrounding parking areas. The empirical credibility of the traffic flow modeling proper is established by calibrating the simulation against observed traffic characteristics such as capacity drop and hysteresis [12]. Agents drive at the maximum feasible speed up to the speed limit and no traffic management devices are modeled.

*Contaminant Plume.* The contaminant is modeled as 1000 kgs of a neutrally-bouyant aerosol released on Highway 110 between W 3^rd^ and W 4^th^ Street with 3.6 m/s prevailing winds from the North at 007° measured 10 m above ground level. Humidity and temperature effects, though implemented in *Tenasi*, were not modeled since they were not expected to materially affect this proof of principle demonstration. Computation of the plume path using an 18 million point grid took approximately 2 days on 100 3.0 Ghz Intel Xeon processors. The time histories of contaminant concentration at ground level were written out for subsequent input into the agent model. The time step used for the plume simulation was approximately an order of magnitude smaller than the agent model which therefore used appropriately sampled information from the more fine-grained CFD simulation.

*Behavioral Assumptions.* There is a large empirical literature on human behavior in crises [13]. Key findings from this literature would be incorporated in a future elaboration of the model. However, to demonstrate the feasibility of the hybrid approach, we simply endow agents with a small number of plausible behavior rules. Specifically, we randomly assign each agent one of three “target” destinations outside the city (intended to represent either exit routes or locations of homes and schools). Once out of buildings, agents attempt to proceed to their assigned destination by the most direct route possible. The rate of agent compliance with “shelter in place” (SIP) directives is taken to be an exogenous parameter of the model. In future extensions, compliance rates could be shaped endogenously by events in the model, as suggested in the empirical literature [14]–[15].

*Exposure to Plume.* Actual dosages of agents exposed to the plume are accumulated as the product of exposure time and local contaminant concentration (scaled for building interiors by the permeability factor). Dosages corresponding to substantial injury and to death respectively are assumed to be 1.e-4 ppm-s and 0.1 ppm-s with no modeling of impaired behavior. This could be included in extensions of the model.

*Cost Metric.* In order to aggregate these effects in the results presented below, we will use a notional cost metric (Table 1). Although the cost metric shown here is notional, it illustrates a potential contribution of hybrid CFD-ABM modeling—the ability to define concrete metrics (based on exposure, for example) for policy effectiveness.

**Table 1:** Cost metric.

Description	Exposure Range(ppm-s)	Cost
Minor Trauma	1.e-6–1.e-4	1,000
Major Trauma	1.e-4–0.1	10,000
Fatal	0.1–2.0	100,000

## Results

Each simulation run represents one realization, or sample path, of a stochastic process. A complete sensitivity analysis would involve a full statistical portrait of model behavior derived from an appropriate number of runs. For purposes of methodological exposition, we simply report ensemble averages over a small number of runs. Observed variance was small.

**Figure 1:**
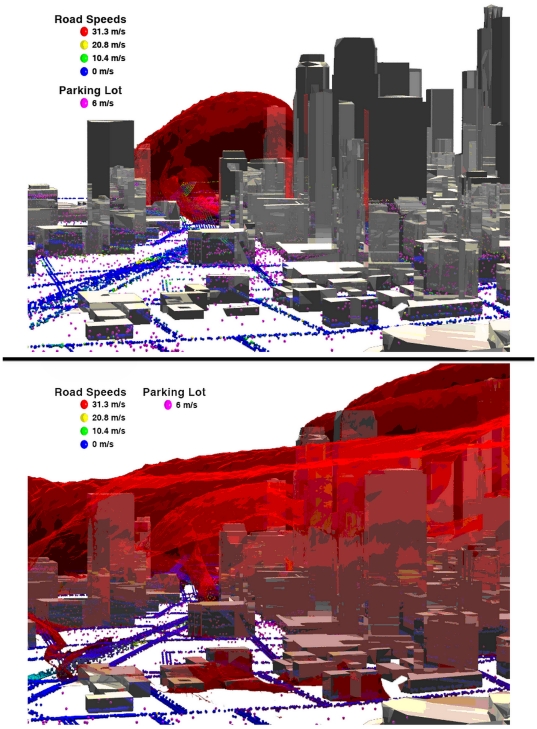
Model snapshot with buildings as reflective polygons, the plume as a translucent red cloud and agents (vehicles) as spheres color-coded by speed. To view a full animation, see: http://www.utc.edu/Research/SimCenter/agent.php.

*Case 1.* In the Base Case neither preparatory nor response policies are in place. Agents leave buildings, adopt their assumed headings, and move according to traffic assumptions. The result is massive congestion, leading to sustained periods in the contaminant plume, high exposure, and high cost. Figure 1 shows screen captures from a simulation of this case (see http://www.utc.edu/Research/SimCenter/agent.php for animated movie).

*Case 2.* In this case, we assume a shelter-in-place (SIP) directive, instructing all individuals in buildings to remain in place. For now, we assume that buildings confer 100% protection from the contaminant, and that compliance with the directive is 100%. People outside the buildings adopt headings and movement rules as in Case 1. SIP has two effects: it protects individuals in buildings, and has a positive externality of reducing congestion. The combined result is that cost is lowered by 99%. However, this result relies on the assumptions of complete protection and complete compliance—which are relaxed in case 3 below.

**Figure 2:**
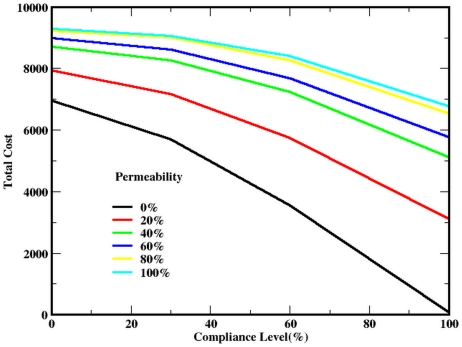
Cost curves as a function of SIP compliance level for varying building permeability.

*Case 3.* Deviations from full compliance produce (nonlinear) increases in congestion and attendant casualties. Even when buildings confer no protection (light blue curve in Figure 2), increase in compliance from 0 to 100% decreases cost by 27% by reducing congestion.

Figure 2 establishes three important, albeit preliminary, points. First, for any fixed level of permeability (indicated by a color), total cost decreases with increasing compliance. This suggests that policies boosting compliance—like prior risk communication and practice exercises—can be effective. Second, at any fixed level of compliance, cost rises with increasing permeability. However, the marginal effect of a unit increase in permeability grows with compliance. That is, the distance between permeability curves is greatest at 100% compliance and lowest at zero compliance. Third, at any fixed level of cost, there are equivalent combinations of permeability and compliance. It is clear that with further computation, the entire tradeoff curve could be generated. Assuming this isoquant to be concave, the economically optimal point could be calculated at any specified budget.

## Discussion

The model introduced here could be applied to evaluate a wide range of other strategies, including: car-pooling, adaptive traffic-aware routing, and staged evacuation, in various combinations.

Our central goal here, however, is methodological: to demonstrate the feasibility of CFD-ABM Plume-Agent Hybrid modeling, and to suggest the powerful ways in which it can be employed for catastrophic event preparedness and response planning. For example, it permits the construction of curves showing the tradeoff between permeability and the SIP compliance level. The latter is shown to have two potential effects: the direct effect of reducing exposure, and the indirect (and highly nonlinear) effect of reducing congestion. Counterintuitively, the latter *alone*—he positive externality of reduced congestion—reduced costs by almost 30% from the Base Case. Thus relatively undemanding measures, including car-pooling, improved routing (e.g., one way outward), some SIP, and egress staging, might be combined to produce dramatically improved outcomes (costs avoided). The model can identify critical thresholds (e.g., the minimum level of SIP) that must be exceeded to avoid catastrophe.

We do not claim to have identified these thresholds (or to have constructed confidence intervals around them), or to have found the optimal solution for Los Angeles. We have developed a novel cross-disciplinary tool allowing one to rigorously do so. This represents a significant advance in evacuation design.

This particular city was chosen because of its obvious importance, and suitability to the exposition. Future research would improve the analysis for LA, and develop the analogous model for other major cities. As noted, the particular scenario in any city is stochastic and would depend on the aerosol, wind field, time of day (rush hour or not), prior planning and exercises, and other factors. However, one could pre-compute a great many scenarios, essentially developing a "petabyte playbook" for major urban centers that could be refined in the actual case. A fast prediction tool for contaminant transport based on reduction/correlation of CFD simulations has been reported in the literature [16]. Indeed, computing is rapidly approaching the point where projections can be made is faster-than-real time and streamed to emergency managers ahead of the plume, allowing routing and other decisions to be adapted as the crisis demands.

## References

[1] Briley WR, Taylor LK, Whitfield DL (2003). High-resolution viscous flow simulations at arbitrary Mach number.. J Comput Phys.

[2] Strelets M (2001). Detached eddy simulation of massively separated flows..

[3] Nichols DS (2006). Aerosol propagation in an urban environment..

[4] Epstein JM, Axtell R (1996). Growing Artificial Societies..

[5] Epstein JM (2006). Generative Social Science..

[6] Longini I, Halloran M, Nizam A, Yang Y, Xu S (2007). Containing a Large Bioterrorist Smallpox Attack: A Computer Simulation.. INT J INFECT DIS.

[7] Axtell R, Dean J, Gumerman G, Swedlund A, Chakravarty S (2007). Population Growth and Collapse in a Multi-Agent Model of the Kayenta Anasazi in Long House Valley.. P Natl Acad Sci USA.

[8] Simon H (1982). Models of Bounded Rationality..

[9] Glimcher PW, Camerer CF, Fehr E, Poldrack RA (2009). Neuroeconomics: Decision Making and the Brain,.

[10] US Geological Survey (2008). The National Map Seamless Server.. http://seamless.usgs.gov.

[11] Marmot AF, Eley J (2000). Office Space Planning: Designs for Tomorrow's Workplace..

[12] Zhang HM, Kim T (2005). A car-following theory for multiphase vehicular traffic flow.. Transp Res Rec B.

[13] McEntire DA (2006). Disaster Response and Recover..

[14] Epstein J, Hammond R, Parker J, Cummings, D (2000). Coupled Contagion Dynamics of Fear and Disease: Mathematical and Computational Explorations.. PLoS One.

[15] Glass T, Schoch-Spana M (2002). Bioterrorism and the People: How to Vaccinate a City Against Panic.. Clin Infect Dis.

[16] Boris J, Fulton JE, Obenschain K, Patnaik G, Young T (2004). CT-Analyst: fast and accurate CBR emergency assessment in Chemical and Biological Sensing V, eds Gardner PJ Proc.. SPIE.

